# *De Novo* Adult Transcriptomes of Two European Brittle Stars: Spotlight on Opsin-Based Photoreception

**DOI:** 10.1371/journal.pone.0152988

**Published:** 2016-04-27

**Authors:** Jérôme Delroisse, Jérôme Mallefet, Patrick Flammang

**Affiliations:** 1 University of Mons—UMONS, Research Institute for Biosciences, Biology of Marine Organisms and Biomimetics, Mons, Belgium; 2 School of Biological & Chemical Sciences, Queen Mary University of London, London, United Kingdom; 3 Catholic University of Louvain-La-Neuve, Marine Biology Laboratory, Place croix du Sud, Louvain-La-Neuve–Belgium; Karlsruhe Institute of Technology, GERMANY

## Abstract

Next generation sequencing (NGS) technology allows to obtain a deeper and more complete view of transcriptomes. For non-model or emerging model marine organisms, NGS technologies offer a great opportunity for rapid access to genetic information. In this study, paired-end Illumina HiSeq^TM^ technology has been employed to analyse transcriptomes from the arm tissues of two European brittle star species, *Amphiura filiformis* and *Ophiopsila aranea*. About 48 million Illumina reads were generated and 136,387 total unigenes were predicted from *A*. *filiformis* arm tissues. For *O*. *aranea* arm tissues, about 47 million reads were generated and 123,324 total unigenes were obtained. Twenty-four percent of the total unigenes from *A*. *filiformis* show significant matches with sequences present in reference online databases, whereas, for *O*. *aranea*, this percentage amounts to 23%. In both species, around 50% of the predicted annotated unigenes were significantly similar to transcripts from the purple sea urchin, the closest species to date that has undergone complete genome sequencing and annotation. GO, COG and KEGG analyses were performed on predicted brittle star unigenes. We focused our analyses on the phototransduction actors involved in light perception. Firstly, two new echinoderm opsins were identified in *O*. *aranea*: one rhabdomeric opsin (homologous to vertebrate melanopsin) and one RGR opsin. The RGR-opsin is supposed to be involved in retinal regeneration while the r-opsin is suspected to play a role in visual-like behaviour. Secondly, potential phototransduction actors were identified in both transcriptomes using the fly (rhabdomeric) and mammal (ciliary) classical phototransduction pathways as references. Finally, the sensitivity of *O*.*aranea* to monochromatic light was investigated to complement data available for *A*. *filiformis*. The presence of microlens-like structures at the surface of dorsal arm plate of *O*. *aranea* could potentially explain phototactic behaviour differences between the two species. The results confirm (i) the ability of these brittle stars to perceive light using opsin-based photoreception, (ii) suggest the co-occurrence of both rhabdomeric and ciliary photoreceptors, and (iii) emphasise the complexity of light perception in this echinoderm class.

## Background

With the publication of the sea-urchin genome acting as a catalyser [1, 2], the number of molecular studies targeting echinoderm species recently exploded [3–20]. However, the molecular information available for non-model echinoderm species remains limited. The development of Next Generation Sequencing (NGS) methods (and RNA-seq in particular) gives access to functional data for non-model species in a faster and cheaper way [21–22]. NGS technologies generate large numbers of reads with high sampling rates of cDNA libraries, providing a deeper and more complete view of transcriptomes [21, 23].

To understand in depth how the ability to perceive light evolved and diversified in eumetazoans, it is crucial to identify the complete suite of genes involved in this phenomenon, as well as their interactions, across a diverse range of species. Eye and photoreception studies have been concentrated on only few model organisms (for review [24]). Despite the advent of NGS technologies and transcriptomic studies, only a small fraction of the currently described “photoreception diversity” is represented in molecular databases. The majority of these molecular resources are derived from the study of insect or crustacean compound eyes and of vertebrate camera type eyes while other phyla–especially those which do not exhibit eyes *stricto sensu*, like echinoderms—have been largely understudied until recently [25–30, 19]. In the context of sensory biology, brittle stars in particular have retained the attention of several morphologists because of their apparent adaptations for light perception [31–34]. Some phototaxic brittle stars indeed exhibit peculiar aboral skeletal microlenses whose proposed function is to focalise light on underlying putative photosensitive structures. However, none of these particular phototaxic brittle star species has been studied at the molecular level. In other ophiuroid species (e.g., *Ophioderma brevispinum*, *Ophiocomina nigra*, and *Amphiura filiformis*), opsins, photosensitive proteins which are involved in both vision and non-visual photoreception (for review [24, 35–36]), have been detected in the arms [28–29, 37]. Recently, 13 putative opsin genes were identified in the draft genome of *A*. *filiformis* [29], suggesting that opsin-based photoreception should be a complex phenomenon in brittle stars.

In this study, we characterised *de novo* arm transcriptomes of two common European brittle stars, *A*. *filiformis* and *Ophiopsila aranea*. While the former is a burrowing species typically found in muddy environment of Swedish fjords, the latter is present under rocks or in crevices of the Mediterranean rocky shores. In the framework of environment perception and particularly the light perception processes, we targeted adult arm tissues in these two brittle star species with contrasted ecology. Indeed, the arms are the organs extending in the water column and, therefore, susceptible to perceive environmental stimuli such as light. Arm transcriptomes were sequenced using HiSeq^TM^ 2000 Illumina technology and functional annotation of the transcriptomes was performed. The presence of molecular pathways related to opsin-based light perception was investigated in the two species. We highlighted opsins and putative phototransduction actors in both transcriptomes, indicating the presence of opsin-based photoreception. Additionally, behavioural experiments were performed on *O*. *aranea* to evaluate its light perception ability. In the case of *A*. *filiformis*, this study complements the study of Delroisse et al. [29]. Finally, the presence of dorsal microlens-like structures [31–34], defined as potential optical structures of ophiuroids, was investigated in both species.

## Method and Materials

### Sample collection and morphological analyses

Specimens of *Amphiura filiformis* (Müller, 1776) were collected at the Lovén Center (Kristineberg, Fiskebäckskil, Sweden) by using a mechanical mud grab at 25–40 m depth in November 2012. Specimens of *Ophiopsila aranea* Forbes, 1843 were collected at the ARAGO biological station (C.N.R.S.) of Banyuls-sur-Mer (France) by SCUBA diving at 20–25m depth during the summer 2013. No specific permissions were required for these locations/activities. The field studies did not involve endangered or protected species.

For scanning electron microscopy (SEM), brittle stars were anaesthetised by immersion in 3.5% w/v MgCl_2_ in filtered seawater. Arms were dissected and their skeletal ossicles were obtained by incubating arm pieces in 10% (v/v) common bleach. Cleaned dorsal plates were rinsed in distilled water, air-dried, mounted on aluminium stubs and coated with gold. They were observed with a JEOL JSM-6100 scanning electron microscope.

### RNA extractions, cDNA library preparation and sequencing

After anesthesia, brittle star arms were dissected and directly frozen in liquid nitrogen. Pieces of arm tissues (from 3 individuals) were then permeabilised in RNA later Ice (Life Technologies) during one night at -20°C following the manufacturer’s instructions and then stored at -80°C until RNA extraction or directly processed for RNA extraction. For both species, total RNA was extracted from adult arms following the Trizol® reagent based method. The quality of the RNA extracts was checked by gel electrophoresis on a 1.2 M TAE agarose gel, and by spectrophotometry using a nanodrop spectrophotometer (LabTech International). The quality of the RNA was also assessed by size-exclusion chromatography with an Agilent Technologies 2100 Bioanalyzer. After treatment of the total RNA with DNase I, magnetic beads with Oligo(dT) were used to isolate mRNAs. The purified mRNAs were then mixed with fragmentation buffer and fragmented into small pieces (100-400bp) using divalent cations at 94°C for 5 minutes. Taking these short fragments as templates, random hexamer primers (Illumina) were used to synthesise the first-strand cDNA, followed by the synthesis of the second-strand cDNA using RNaseH and DNA polymerase I. End reparation and single nucleotide A (adenine) addition were performed on the synthesised cDNAs. Next, Illumina paired-end adapters were ligated to the ends of these 3’ adenylated short cDNA fragments. To select the proper templates for downstream enrichment, the products of ligation reaction were purified on a 2% agarose gel. The cDNA fragments of about 200bp were excised from the gel. Fifteen rounds of PCR amplification were carried out to enrich the purified cDNA template using PCR primer PE 1.0 and 2.0 (Illumina Inc., San Diego, CA, USA) with phusion DNA polymerase. Finally, the cDNA library was constructed with 200bp insertion fragments. After validation on the Agilent Technologies 2100 Bioanalyzer, the library was sequenced using Illumina HiSeq^TM^ 2000 (Illumina Inc., San Diego, CA, USA), and the workflow was as follows: template hybridisation, isothermal amplification, linearisation, blocking, sequencing primer hybridisation, and first read sequencing. After completion of the first read, the templates are regenerated *in situ* to enable a second read from the opposite end of the fragments. Once the original templates are cleaved and removed, the reverse strands undergo sequencing-by-synthesis. High-throughput sequencing was conducted using the Illumina HiSeq^TM^ 2000 platform to generate 100-bp paired-end reads. cDNA library preparation and sequencing were performed at Beijing Genomics institute (BGI, Hong Kong) according to the manufacturer’s instructions (Illumina, San Diego, CA). After sequencing, raw image data were transformed by base calling into sequence data, which were called raw reads and stored in the fastq format. Transcriptome quality was checked using the FastQC software [38].

### *De novo* transcriptome assembly

*De novo* transcriptome assembly was performed separately from *A*. *filiformis* and *O*. *aranea* reads. Before the transcriptome assembly, the raw sequences were filtered to remove the low-quality reads. The filtration steps were as follows: 1) removal of reads containing only the adaptor sequence; 2) removal of reads containing over 5% of unknown nucleotides ‘‘N”; and 3) removal of low quality reads (those comprising more than 20% of bases with a quality value lower than 10). The remaining clean reads were used for further analysis. Quality control of reads was accessed by running the FastQC program (http://www.bioinformatics.babraham.ac.uk/projects/fastqc/).

Transcriptome *de novo* assembly was carried out with short paired-end reads using the Trinity software [39] (version release-20121005; min_contig_length 100, group_pairs_distance 250, path_reinforcement_distance 95, min_kmer_cov 2). Trinity partitions the sequence data into many individual de Bruijn graphs, each representing the transcriptional complexity at a given gene or locus, and then processes each graph independently to extract full-length splicing isoforms and to tease apart transcripts derived from paralogous genes. After Trinity assembly, the TGI Clustering Tool (TGICL, [40] followed by Phrap assembler (http://www.phrap.org) were used for obtaining distinct sequences. These sequences are defined as unigenes. Unigenes can either form clusters in which the similarity among overlapping sequences is superior to 94%, or singletons that are unique unigenes.

As the length of sequences assembled is a criterion for assembly success, we calculated the size distribution of both contigs and unigenes. To evaluate the depth of coverage, all usable reads were realigned to the unigenes using SOAP aligner with the default settings [41]. Additionally, the completeness of the transcriptomes was evaluated using tBLASTn search against the 248 “Core Eukaryotic Genes” [42] (http://korflab.ucdavis.edu/datasets/cegma/)

BLASTx alignments (E-value threshold < 1e^-5^) between unigenes and protein databases like NCBI nr (http://www.ncbi.nlm.nih.gov/), Swiss-Prot (http://www.expasy.ch/sprot/), KEGG (http://www.genome.jp/kegg/) and COG (http://www.ncbi.nlm.nih.gov/COG/) was performed, and the best aligning results were used to identify sequence direction of unigenes. When results from different databases are conflicting, the priority order nr, Swiss-Prot, KEGG, and COG was followed to decide on sequence direction for unigenes. When a unigene was unaligned in all of the above databases, the ESTScan software (v3.0.2) [43] was used to decide on its sequence direction. ESTScan produces a nucleotide sequence (5’–3’) direction and the amino sequence of the predicted coding region.

For both transcriptomes, unigene expression was evaluated using the “Fragments per kilobase of transcript, per million fragments sequenced” (FPKM) method. The FPKM value is calculated following the specific formula $FPKM=\frac{10^{6}C}{NL/10^{3}}$ where C is the number of fragments showed as uniquely aligned to the concerned unigene, N is the total number of fragments that uniquely align any unigene, and L is the base number in the coding DNA sequence of the concerned unigene. The FPKM method integrates the influence of different gene length and sequencing level on the calculation of gene expression.

### Functional gene annotations

**Fig 1:**
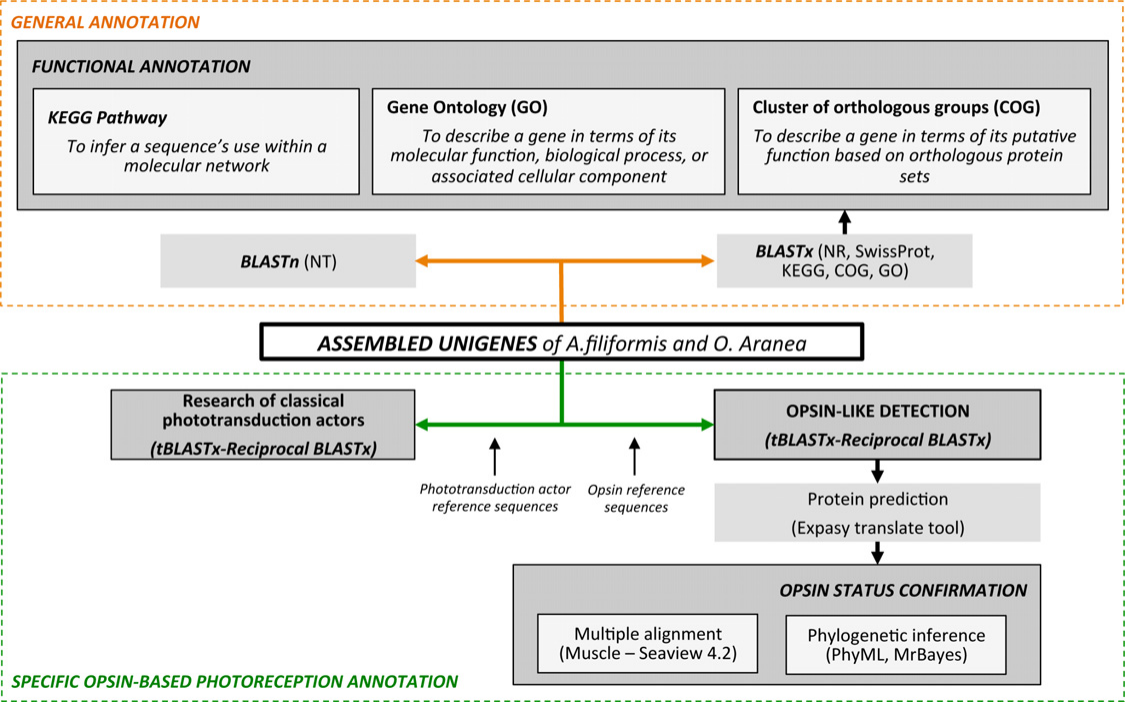
Annotation analysis pipeline of the transcriptomes of *A*. *filiformis* and *O*. *aranea*.

Following the pipeline described in the Fig 1, all unigenes were used for homology searches against various protein databases such as NCBI nr, Swissprot, COG, and KEGG pathway with the BLAST + software (BLAST x, E-value **<** 1e^-5^). Best results were selected to annotate the unigenes. When the results from different databases were conflicting, the results from nr database were preferentially selected, followed by Swissprot, KEGG and COG databases. Unigene sequences were also compared to nucleotide databases nt (non-redundant NCBI nucleotide database, E-value < 1e^-05^, BLASTn, http://www.ncbi.nlm.nih.gov/).

In order to estimate the number of assembled transcripts that appear to be nearly full-length, the unigenes were aligned against all known proteins from nr database and the numbers of unique top matching proteins (E-value < 1e^-05^) that align across more than a certain percentage of the reference protein length were counted.

To further annotate the unigenes, the Blast2GO program [44–45] was used with nr annotation to get GO annotation according to molecular function, biological process and cellular component ontologies (http://www.geneontology.org/). Blast2GO is widely recognised as a GO annotation software. Gene Ontology (GO) is an international standardised gene functional classification system which offers a dynamically updated and controlled vocabulary and a strictly defined concept to comprehensively describe properties of genes and their products in any organism. After getting GO annotation for every unigenes, the WEGO software [46] was used to establish GO functional classification for all unigenes and to highlight the distribution of gene functions.

The unigene sequences were also aligned to the COG database to predict and classify possible functions. COG is a database in which orthologous gene products are classified. Every protein in COG is assumed to have evolved from an ancestor protein, and the whole database is built on coding proteins with complete genome as well as system evolution relationships of bacteria, algae and eukaryotic organisms. Finally pathway assignments were performed according to the KEGG pathway database [47–48]. The KEGG pathway database records networks of molecular interactions in cells, and variants of them specific to particular organisms. Potential molecular pathways involving predicting unigenes can be highlighted from KEGG pathway annotations. The top 15 KEGG pathways were identified in both transcriptomes. Additionally, specific KEGG pathways related to light perception were targeted.

### Opsin genes in *O*. *aranea*

Retrieval of opsin sequences in the *Af* transcriptome was previously reported [29] on the basis of the Illumina data described in the present study. For *O*. *aranea*, local tBLASTn (v2.2.26) [49] searches were used to identify the opsin mRNA sequence expressed in the *Oa* transcriptome using reference opsin sequences as queries (*S*. *purpuratus*, *A*. *filiformis* and other metazoans opsins, S1 Table, see also [29]). Candidate matches were used as queries in a reciprocal BLASTx (v2.2.25) search against online databases (NCBI) in order to highlight sequences with high similarity to opsins. *In silico* translation (Expasy translate tool, [50]) was performed on the opsin-like sequences retrieved from the *Oa* transcriptome. Sequence alignments allowed the identification of *bona fide* opsin sequences after transmembrane helix and Schiff base detection. Secondary structure prediction–in particular of transmembrane helices–was done using the MENSAT online tool [51]. A multiple amino-acid alignment of the putative opsins was performed using Seaview (v4.2.12) [52] and muscle algorithm [53]. Aligned residues were highlighted by similarity group conservation (defined by the software) and similarity comparisons were calculated in Mega v6 [54–55]. Sequence alignments also made it possible to identify opsin characteristic features such as the lysine residue involved in the Schiff base linkage, the counterion, the amino acid triad present in the helix involved in the G protein contact, or putative disulfide bond sites. The predicted molecular weight of the opsins was calculated using the “Compute pI/Mw tool” on the ExPASy Proteomics Server [50].

A phylogenetic tree of echinoderm opsins, including the new opsin sequences from *O*. *aranea*, was constructed using truncated sequences (i.e., conserving the conserved 7TM core of the protein and discarding opsin extremities to avoid unreliably aligned regions). A sequence of a non-opsin GPCR (i.e. melatonin receptor) was chosen as outgroup following recent studies [56–58, 29]. A maximum likelihood phylogeny was constructed using the PHYML tool [59–60] from the SeaView 4.2.12 software [52], which allows for the fast estimation of large data sets within a maximum likelihood (ML). The WAG (Wheland and Goldman) model of protein evolution, predicted as the best fitting ML model using Mega v56 [61], was used for ML analysis. Branch support values were estimated as bootstrap proportions from 500 PhyML bootstrap replicates. We also performed a Bayesian analysis with MrBayes 3.2 [62] using the GTR+G model based on recent opsin studies [57–58, 28–29, 19]. Four independent runs of 6,000,000 generations were performed, until a standard deviation value inferior to 0.01 is reached [62, 28–29]. Sequences used in the phylogenetical analyses are listed in the S1 Table.

### Phototransduction actor genes in *A*. *filiformis* and *O*. *aranea*

We searched for and identified putative homologues to proteins involved in both ciliary and rhabdomeric phototransduction pathways in *Af* and *Oa* transcriptomes. Reference phototransduction protein sequences [63–67] (S2 Table) were used as queries in tBLASTn searches against the set of *A*. *filiformis* and *O*. *aranea* unigenes. A reciprocal best BLASTx search was performed using the top unigenes against the NCBI non-redundant protein database.

### Behavioural study of light perception in *O*. *aranea*

In order to test brittle star phototactic behaviour, light perception experiments were performed on adult specimens of *O*. *aranea*. Monochromatic LED lamp (1W) illuminations were used and their spectra were first evaluated with a minispectrometer (Hamamatsu Photonics K.K. TM–VIS/NIR: C10083CA, Hamamatsu-City, Japan). Experiments were conducted in an elongated aquarium (50 x 15 cm and 12 cm high) with specific light illumination (1W LED dimmed by neutral density filter ND5 allowing 20% of the remaining light; white light; green light: λmax = 515nm, half peak bandwidth HBW≈41nm; blue light: λmax = 464nm, HBW≈36nm; red light: λmax = 632nm, HBW≈32nm; yellow light: λmax = 575nm, HBW ≈20nm; and a no-light control observed under infrared illumination) on one side of the aquarium. At the time T0, one individual is placed at the center of the aquarium. The distance travelled by the brittle star in the aquarium and its speed (based on time and distance) are measured during 2-minute sessions (non-directional displacement). To account for directionality, the measured distances were converted to positive (+) or negative (−) values depending on whether the brittle star was moving away (by convention: +) from or toward (-) the light source, respectively (directional displacement). The corresponding speeds are then also positive or negative. Plus and minus were arbitrarily set for the measurements under the no-light condition. For both non-directional and directional displacements (distances and speeds), medians of all colour treatments were compared to the dark control experiment in which no illumination was used (non-parametric Mann-Whitney test). Statistical analyses were performed using GraphPad Prism 5.0 (GraphPad Software, San Diego, CA, USA; www.graphpad.com).

## Results and Discussion

### Morphology and photobehaviour in *A*. *filiformis* and *O*. *aranea*

**Fig 2:**
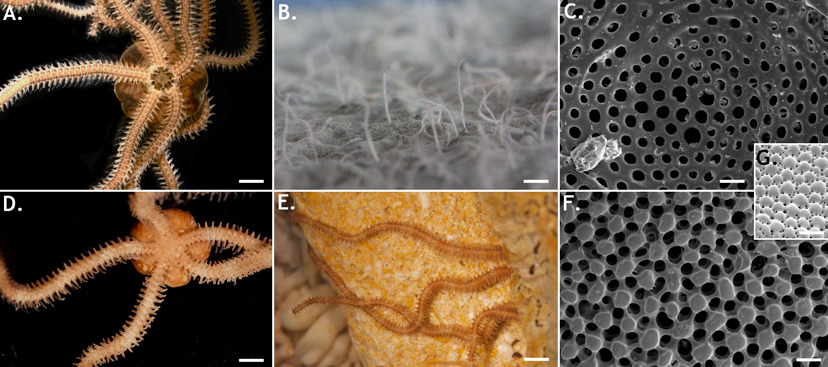
**The brittle stars *Amphiura filiformis* (A-C) and *Ophiopsila aranea* (D-F).** (A) individual in oral view. (B) Feeding arms protruding out of the sediment, in aquarium. (C) Central area of a dorsal arm plate. (D) Individual in aboral view. (E) Feeding arms out of the coralligen, in situ. (F) Central area of a dorsal arm plate showing expanded peripheral trabeculae. (G) Central area of a dorsal arm plate of *Ophiocoma wendtii* showing microlenses, from [34] (Scale bars: A: 900 μm, B: 1cm, C: 30 μm, D: 5mm, E: 20mm, F: 30 μm, G: 70 μm).

Like in all known brittle stars, the arms of *A*. *filiformis* and *O*. *aranea* consist of a repeated series of well-defined skeletal structures [68–71]. The major skeletal pieces are the dorsal, ventral and paired lateral arm plates. Additionally one vertebral ossicle (the so-called ambulacral ossicle) is enclosed in each segment. The plates consist of two main parts: the stereom which is an echinoderm-specific three dimensional network of high-magnesium calcite trabeculae and the stroma which consists of soft tissues filling the holes and interstices of the stereom [68,70–72]. Observed by scanning electron microscopy, the dorsal arm plates of *O*.*aranea* bear hundreds of microscopic knobs described by [33,34] as enlarged peripheral trabeculae (EPT) (Fig 2F). These structures are absent from the dorsal arm plates of *A*. *filiformis* (Fig 2C). *O*. *aranea* EPT are rounded and smooth and measure between 25 and 35 μm in diameter (Fig 2F). Even if these structures perfectly match with EPT described in *Ophiocoma wendtii* (Fig 2G), in *O*. *aranea* the pores between EPT appeared larger and the globular aspect of the EPT is amplified.

Behavioural experiments were performed on adult individuals of both species using different artificial light sources. In the brittle star *A*. *filiformis*, no displacement was observed (personal observations), indicating that this species does not exhibit any escape behavior upon light illumination. However, the burrowing ecology of *A*. *filiformis* is limiting for the type of behavioural test used in the present study and could explain the lack of responsiveness to light exposure. It is known that, in this species, arm feeding activity is directly linked to the ambient light [73] and, a blue-green light sensitivity was recently highlighted in [29] using another behavioural approach in which the brittle stars could burrow in the sediment.

**Fig 3:**
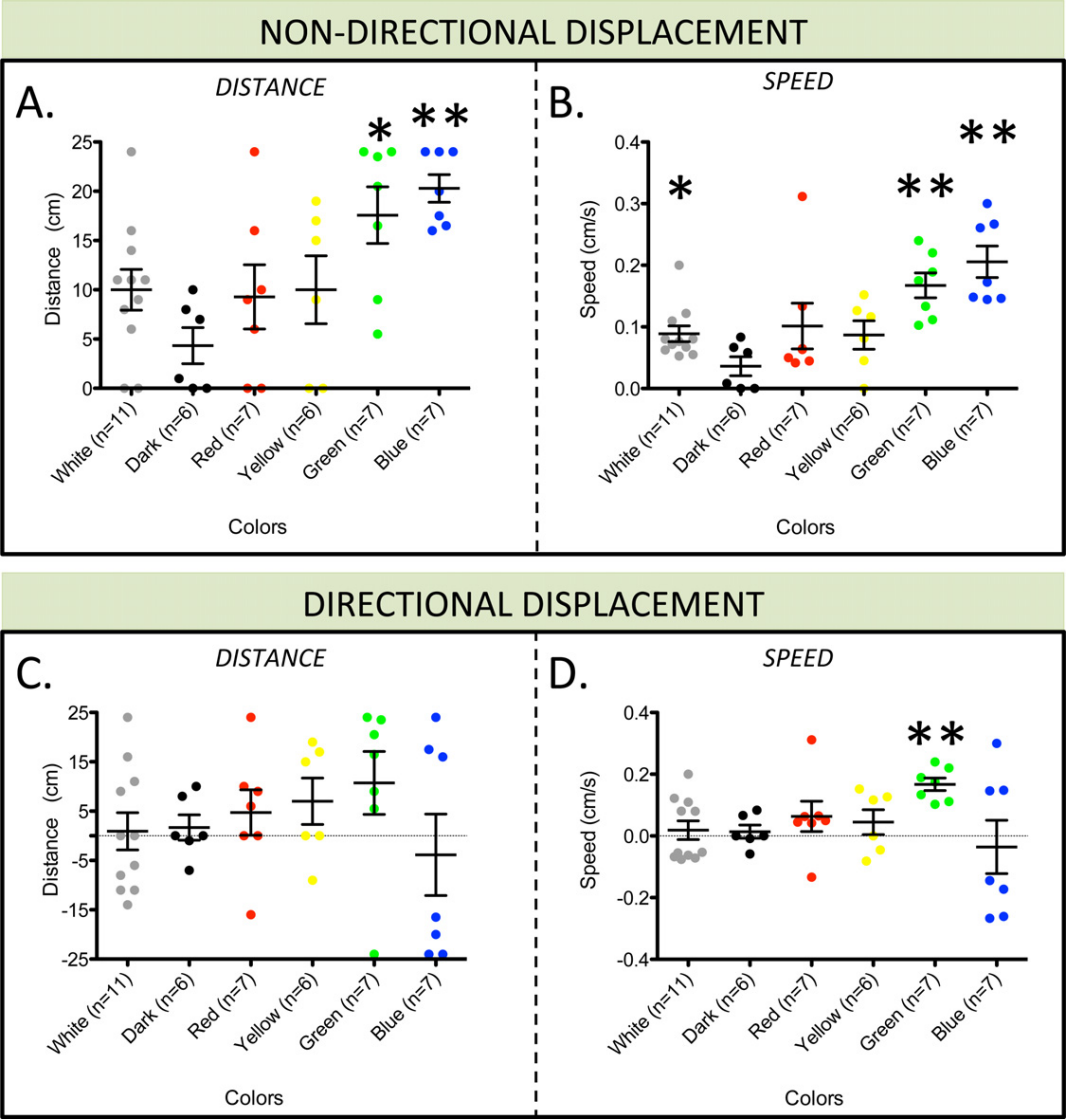
Phototaxis in *Ophiopsila aranea*. (A-B) Dotplot graph of absolute distance span (A) and speed (B) as calculated from 0 position for each different light conditions. (C-D) Dotplot graph of directional distance span (C) and speed (D) as calculated from 0 position for each different light conditions. Measured distances were taken as positive (+) or negative (−) depending upon whether the brittle star was moving away (+) or toward (-) from the light source. Standard error of the mean is shown for each treatment.

In *O*. *aranea*, a clear phototaxis was demonstrated upon illumination with maximal reaction for blue and green colours (464 nm and 515 nm, Mann-Whitney Test, p<0.05 for both treatments using non-directional distances and speeds) (Fig 3A and 3B). Additionally, a negative phototactic response was detected for green light only (Mann-Whitney Test, p-value = 0.0034 using directional speed) (see directional speed comparisons on Fig 3D). Considering the green shifted ambient light of coastal environments (as opposed to blue ambient light of open water environments), a high sensitivity to green light appears advantageous. The distinct observations made for green and blue light treatments are difficult to discuss without additional experiments in which various light intensities and wavelengths could be tested. In the frame of this study, a blue/green light sensitivity was clearly highlighted for *O*. *aranea*. A weak response to red and yellow light is suspected even though no significant differences are detected with the no-light control (see S3 Table for the complete statistical analyses). Similarly to what was observed in other echinoderms, these brittle stars react to illumination by a fast displacement (average speed of 3.4 ±1.09 cm/min, n = 16; pooled data for green and blue lights). As a reference, an average speed of around 4 cm/min was observed by [27] using a similar device on the sea urchin *S*. *purpuratus*. Comparisons between species exhibiting contrasted locomotion modes (and different sizes) is however awkward. Sea urchins use their spines and tube feet for locomotion [74] whereas brittle stars use their five arms to apply forces to the substratum [75]. In a comparative view, the average speed of the fast-moving brittle star species *Ophiocomina nigra* was measured at around 45 ±4.8 cm/min (J.D., data not shown, in same conditions).

### Illumina sequencing and Reads assembly

In order to characterise the transcriptomes of *A*. *filiformis* and *O*. *aranea*, mRNAs were extracted from the arms of several individuals, converted to cDNAs, and sequenced using Illumina pair-end sequencing technology (HiSeq™ 2000, BGI tech, Hong Kong). In *A*. *filiformis*, 48,699,894 raw reads with a length of 100bp were generated from a 200bp insert library. After stringent quality checking and data cleaning (removing redundant reads, filtering reads of low quality, trimming low-quality ends and removing adapters), the remaining 46,168,240 high quality reads were used to assemble the transcriptome. In *O*. *aranea*, 47,700,906 raw reads were generated. Following the previously mentioned cleaning steps, 45,158,822 clean reads were used for analysis. In both transcriptomes, Q20 percentages (base quality higher than 20) were superior to 97%. The N percentage which indicates the percentage of nucleotides which could not be sequenced was estimated to 0%. The GC percentage was 40.82% for the *Af* transcriptome and 41.07% for the *Oa* transcriptome. Raw reads were deposited in the NCBI Short Read Archive (SRA) under the accession numbers SRX660504 for *A*. *filiformis* and SAMN03166075 for *O*. *aranea*. *A*. *filiformis* and *O*. *aranea* unigene fasta files are included as supplementary data (S1 and S2 Files). Sequencing output statistics are presented in Table 1.

**Table 1:** Description of the output sequenced data for the adult arm transcriptomes of *Amphiura filiformis* and *Ophiopsila aranea*.

***Species***	***Amphiura filiformis***		***Ophiopsila aranea***	
***Total Raw Reads***	48,699,894		47,700,906	
***Total Clean Reads***	46,168,240		45,158,822	
***Total Clean Nucleotides (nt)***	4,616,824,000		4,515,882,200	
***Q20 percentage***	98.09%		97.66%	
***GC percentage***	40.82%		41.07%	
	***Contigs***	***Unigenes***	***Contigs***	***Unigenes***
***Total Number***	457,352	136,387	361,854	127,324
***Total Length (bp)***	90,186,310	62,642,665	91,023,564	73,908,927
***Mean Length (bp)***	197	459	252	580
***N50 (bp)***	220	502	312	763
***Distinct clusters***	/	44,310	/	34,254
***Distinct singletons***	/	92,077	/	93,070

The Q20 percentage is the proportion of clean reads with a quality value larger than 20. The GC percentage is the proportion of guanidine and cytosine nucleotides among total nucleotides.

The trinity assembler software, specifically developed for using of NGS short-read sequences [39], was employed for both *de novo* assemblies of the paired-end high quality reads. According to the overlapping information of high-quality reads, 457,352 contigs were generated for the *Af* transcriptome and 361,854 contigs for the *Oa* transcriptome. The average contig length was 197 bp in the former and 252 bp in the latter. The unigene N50 was 502 bp for *A*. *filiformis* and 763 bp for *O*. *aranea*. Contig and unigene N50 values are relatively low but similar or higher than N50 values observed in previous *de novo* RNA-seq analyses performed on ophiuroids [5–6, 19–20].

**Fig 4:**
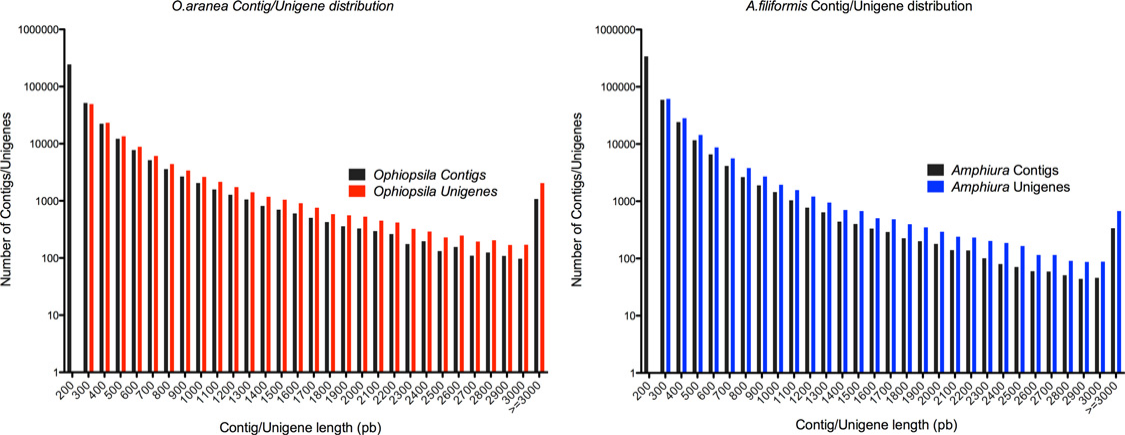
Distributions of contigs and unigenes sizes in Af and Oa transcriptomes. The length of contigs and unigenes ranged from 200 bp to more than 3,000 bp. Each range is defined as follows: sequences within the range of X are longer than X bp but shorter than Y bp.

Using paired-end joining and gap filling, contigs were further assembled into 136,387 unigenes, including 44,310 clusters and 92,077 singletons, with a mean length of 459 bp for the *Af* transcriptome. For the *Oa* transcriptome, 127,324 unigenes with a mean size of 580 bp, including 34,254 clusters and 93,070 singletons, were generated. The size distribution of contigs and unigenes is shown in Fig 4 and numerical data are summarised in Table 1.

**Fig 5:**
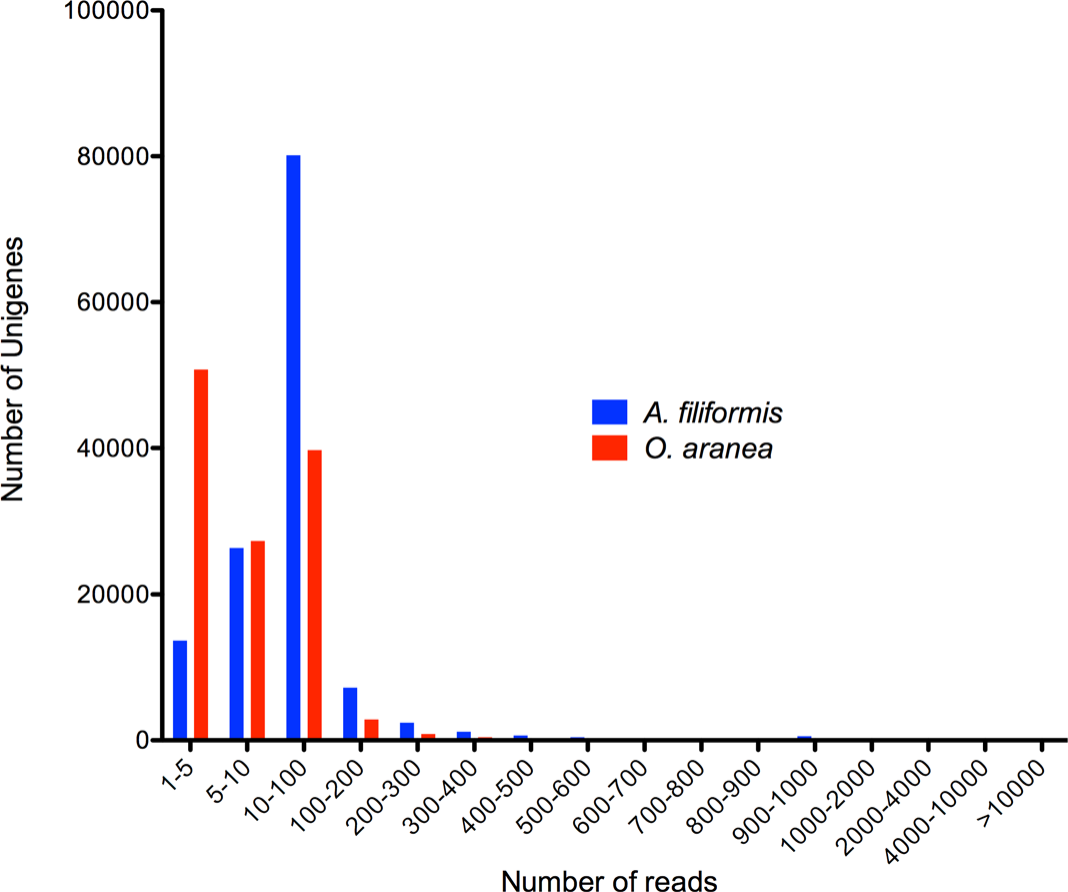
Distribution of the assembled Af and Oa unigenes in function of the number of reads to which they can be aligned. The x-axis represents the « number of reads » classes.

To evaluate the read coverage of the assembled unigenes, all the usable sequencing reads were realigned to the unigenes. More than 89% of *A*. *filiformis* unigenes and more than 58% of *O*. *aranea* unigenes realigned with more than 5 reads (Fig 5).

The completeness of the transcriptomes were also evaluated using tBLASTn search for the 248 “Core Eukaryotic Genes” [42] (http://korflab.ucdavis.edu/datasets/cegma/). A total of 243 (98%) and 242 (98%) of these highly conserved CEGs were detected in the *Af* and Oa transcriptomes, respectively (E-value < 1e^-5^).

Sequence orientation of all unigenes was predicted via ESTscan or the Blast local alignment search tool (E-value < 1e^-5^) in the NCBI database of non-redundant protein (Nr), the Swiss-Prot protein database, the Kyoto Encyclopedia of Genes and Genomes (KEGG) database, and the Clusters of Orthologous Groups (COG) database. In *A*. *filiformis* and *O*. *aranea*, sequence orientations were predicted for 38,967 and 33,951 unigenes, respectively, corresponding to 28.6% and 26.7% of total unigenes.

### Functional annotation of the brittle star transcriptomes

**Fig 6:**
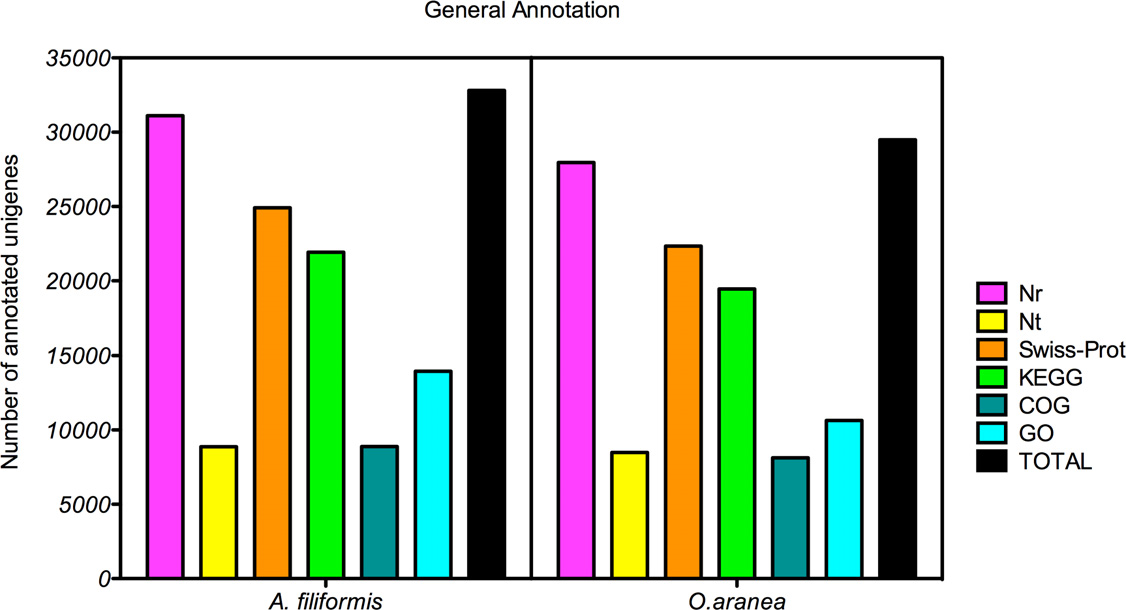
Distribution of annotation results. Unigenes of *Amphiura filiformis* and *Ophiopsila aranea* were annotated using the nr, nt, Swiss-Prot, KEGG, COG and GO databases (see text for details).

For annotation of the assembled unigenes, blast sequence similarity searches were conducted in the nr and nt databases, the COG and GO databases, the Swiss-Prot database, and the KEGG database (E-value threshold of 1e^-5^). Out of the 136,387 *A*. *filiformis* unigenes, 31,109 (22.8%), 8,874 (6.5%), 24,912 (18,2%), 21,931 (16%), 8,880 (6,5%) and 13,950 (10,2%) showed significant similarity to known proteins/genes in the nr, nt, SwissProt, KEGG, COG and GO databases, respectively (Fig 6). For *O*. *aranea*, out of the 127.324 unigenes, 27,959 (22%), 8,490 (6.7%), 22,338 (17.5%), 19,452 (15.3%), 8,132 (6.4%) and 10,643 (8.4%) showed significant similarity to known proteins/genes in the same databases. In total, 32,815 (24%) and 24,490 (19.2%) unigenes were annotated in *A*. *filiformis* and *O*. *aranea*, respectively. Annotation statistics are summarised in the Fig 6. Burns *et al*. [6] obtained a similar gene prediction percentage of 19% for the brittle star *Ophionotus victoriae* (Ophiuroidea). The main reason for these low rates is probably the lack of large-scale genomic resources for the genera *Amphiura and Ophiopsila* and other evolutionary related ophiuroids. Moreover short-sized Unigenes can increase the difficulty of gene identification. Zhou *et al*. [4] obtained higher percentage of gene prediction for the sea cucumber *Apostichopus japonicus* (Holothuroidea), which is explained by the evolutionary proximity between sea cucumbers and sea urchins [17] for which a genome is available. However, obtained annotation rates are comparable to those reported in other studies in which high throughput sequencing technology has been used for *de novo* transcriptome assembly in non-model species [3, 76–80].

**Fig 7:**
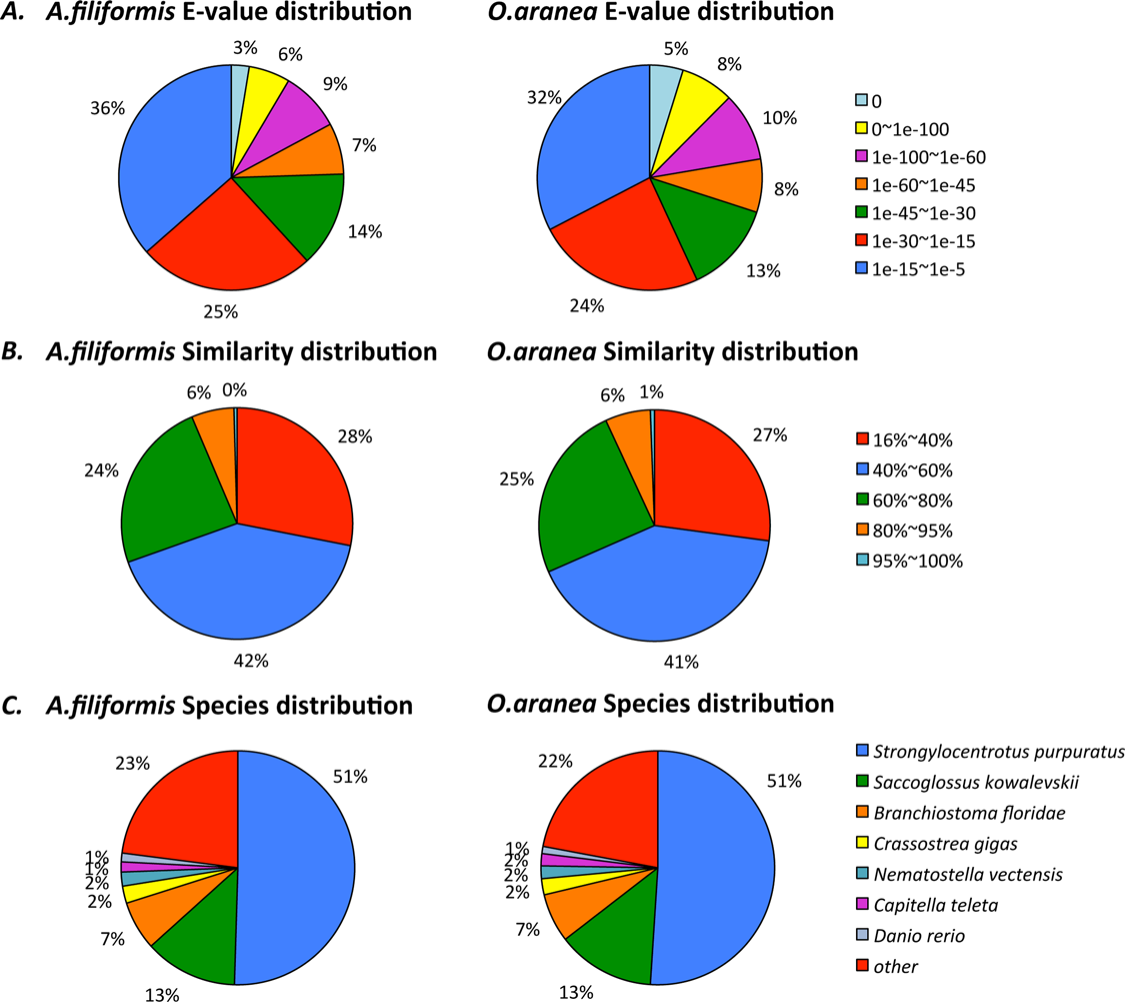
(A) E-value distributions, (B) similarity distributions and (C) species distributions of the top BLAST hits for all unigenes from Af and Oa transcriptomes in the nr database.

The annotation success was estimated by plotting the distributions of annotation E-values and similarity results for the nr database comparison. E-value distributions are presented in Fig 7A. They revealed that 24.4% of the unigenes (7615 unigenes) in the *Af* transcriptome showed significant homology to known proteins (E-value lower than 1e^-45^) against 29.9% (8381 unigenes) in the *Oa* transcriptome. E-value distributions are highly similar in the two transcriptomes with, for example, around 35% of E-values ranging from 1e^-15^ to 1e^-5^ (minimal similarity) in both cases. Similarity distributions are presented in Fig 7B. For both brittle star transcriptomes, between 30 and 35% of the mapped sequences have a similarity greater than 60% with known proteins. Proteins showing similarity to brittle star translated unigenes originate predominantly from the same three species, *S*. *purpuratus*, *Saccoglossus kowalevskii and Branchiostoma floridae*, as expected following their phylogenetic proximity (Fig 7C). A maximal similarity (51%) with the sea urchin *S*. *purpuratus* was observed for both transcriptomes. This was expected given that *S*. *purpuratus* is the only echinoderm that has undergone whole genome sequencing to date and the paucity of ophiuroid genes in GenBank.

Blast results against the nr database were also used to estimate the number of assembled transcripts that appear to be nearly full-length (see S1 Fig). For the *A*. *filiformis* transcriptome, 9% of the unigenes appeared to be aligned by more than 90% in length with the corresponding protein from nr database, 17% appeared to be aligned by more than 60% and 36% appeared to be aligned by more than 30%. For the *O*. *aranea* transcriptome, no unigene appeared to be aligned by more than 90% in length with the corresponding protein from nr database, 0% appeared to be aligned by more than 60% and 38% appeared to be aligned by more than 30%.

The FPKM method was used to estimate gene expression in both *Af* and *Oa* transcriptomes. The 10 most expressed unigenes, predicted to code proteins (BLASTx) are shown in Table 2. Ribosomal proteins constitute the main part of the top 10 expressed unigenes in both species indicating a high ribosomal activity and protein translation in the arms of this species. The mitochondrial gene Cytochrome oxidase linked to energetic metabolism is also highly expressed in *Amphiura*.

**Table 2:** BLASTx of the 10 most expressed unigenes in the arm transcriptomes of *Amphiura filiformis* and *Ophiopsila aranea*.

Unigene ID	TOP 10 most expressed mRNA (FPKM)*	E-value
**Af transcriptome**		
CL20775.Contig1	cytochrome c oxidase subunit I [*Amphipholis squamata*]	0.0
Unigene64582	PREDICTED: similar to ribosomal protein S15e [*Tribolium castaneum*]	4,00E-70
CL3024.Contig1	ubiquitin [*Ostrea edulis*]	3,00E-21
Unigene68129	actin related protein 1 [*Strongylocentrotus purpuratus*]	6,00E-103
Unigene68410	ferritin [*Holothuria glaberrima*]	9,00E-73
Unigene64652	vacuolar sorting protein [*Culex quinquefasciatus*]	7,00E-08
Unigene63757	PREDICTED: 40S ribosomal protein S12-like isoform 1	6,00E-66
Unigene46657	PREDICTED: ribosomal protein L21-like [*Saccoglossus kowalevskii*]	4,00E-63
Unigene63241	PREDICTED: ribosomal protein L13-like [*Saccoglossus kowalevskii*]	1,00E-85
Unigene69766	PREDICTED: myosin regulatory light polypeptide 9-like [*Strongylocentrotus purpuratus*]	1,00E-70
**Oa transcriptome**		
Unigene27354	60S acidic ribosomal protein P1 [*Danio rerio*]	1,00E-38
Unigene40440	PREDICTED: ribosomal protein S10-like [*Saccoglossus kowalevskii*]	4,00E-68
Unigene14567	PREDICTED: 40S ribosomal protein S26-like [*Strongylocentrotus purpuratus*]	5,00E-58
CL6099.Contig1	PREDICTED: 60S ribosomal protein L13a-like isoform 3 [*Strongylocentrotus purpuratus*]	2,00E-80
Unigene37890	PREDICTED: 60S ribosomal protein L32-like [*Strongylocentrotus purpuratus*]	2,00E-55
Unigene37889	PREDICTED: ribosomal protein L35-like [*Saccoglossus kowalevskii*]	4,00E-49
Unigene47692	hypothetical protein BRAFLDRAFT_97880 [*Branchiostoma floridae*]	1,00E-133
Unigene31007_Oa	40S ribosomal protein S24 [*Saccoglossus kowalevskii*]	4,00E-59
Unigene42722_Oa	ribosomal-like protein [*Phragmatopoma lapidosa*]	3,00E-76
Unigene40439_Oa	60S ribosomal protein L23 [*Aedes aegypti*]	6,00E-69

^*^ Fragments per kilobase of transcript, per million fragments sequenced.

### Functional classification by “Gene Ontology” and “Clusters of Orthologous Groups”

**Fig 8:**
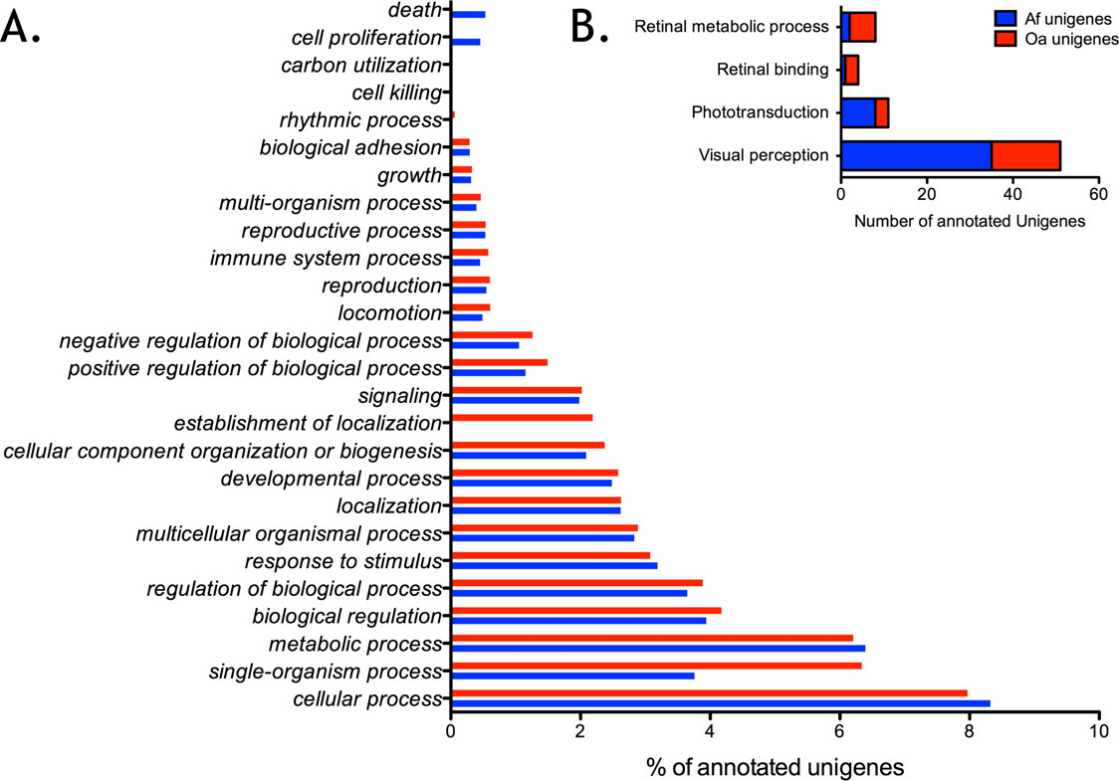
Gene ontology classification of assembled unigenes from the arm transcriptomes of *Amphiura filiformis* and *Ophiopsila aranea*. In both transcriptomes the assignments to the “biological process” category made up the majority of the annotations (Af: 46,641; 47.5%; Oa: 47046, 52.5%) followed by the “cellular component” category (Af: 34,337; 34.9%; Oa: 29,433, 32.9%) and “molecular function” category (Af: 17,280; 17.6%; Oa: 13061, 14.6%). A. The results of the “biological process” category are presented in percentages of the total annotated unigenes. B. The results relative to retinal metabolic process (GO 0042574), retinal binding (GO 0016918), phototransduction (GO 0007602) and visual perception (GO 0007601) are shown.

Gene ontology, defined as a standardised gene functional classification system, constitutes a useful tool to annotate and analyse the functions of a large number of genes/proteins. On the basis of the nr annotation, the Blast2GO software [44–45] was first used to obtain GO functional classifications of the assembled unigenes. For the *Af* transcriptome, 13,950 unigenes with BLAST matches to known proteins were assigned to the three ontologies (molecular function, cellular component and biological process) of GO classes with, in total, 98,258 functional terms. For the *Oa* transcriptome, 10,643 unigenes were assigned to a total of 89,540 functional GO terms. For *A*. *filiformis*, under the “biological process” category, “cellular processes” (8,175; 17.5% of the biological process total) and “metabolic processes” (6,282; 13.5% of the biological process total) were prominently represented, indicating logically that some important metabolic activities and cell processes occur in brittle star arms (Fig 8A). For *O*. *aranea*, “cellular processes” (7,136; 15.2% of the biological process total) and “single-organism processes” (5,675; 12% of the biological process total) were the two first detected categories while “metabolic processes” was the third (5,557; 11.8% of the biological process total). However the distribution across the three ontologies is highly similar between the two transcriptomes (Fig 8A). As also observed in other echinoderm (and non-echinoderm) species, under the classification of molecular function, binding and catalytic activity were separately the first and second largest categories in both transcriptomes [3,81,82]. For the “cellular component” ontology, four categories, cell, cell part, organelle and organelle part, accounted for approximately 70% of the cellular components whereas only a few unigenes were assigned to extracellular region, membrane or synapse, for example, in both transcriptomes. Compared with GO category assignment of *S*. *purpuratus* and *A*. *japonicus* transcriptomes, dominant categories are coincident in the GO classes [3,19,81,82].

Specific GO categories related to the light perception process, including “Visual perception”, “Phototransduction”, “Retinal binding” and “Retinal metabolic process”, were targeted in *Af* and *Oa* transcriptomes. These four GO allowed the respective identification of 35, 8, 1 and 2 gene candidates in *A*. *filiformis*, and 16, 3, 3 and 6 gene candidates in *O*. *aranea*, indicating the clear presence of those processes in both species (Fig 8B).

The annotated sequences were used to search for genes in the COG classification. The Clusters of Orthologous Groups (COGs) [83,84] is a database in which orthologous gene products are classified. Each cluster contains proteins or groups of paralogues from different lineages. Orthologues typically have the same function, allowing transfer of functional information from one member to an entire COG. This relation automatically yields a number of functional predictions for poorly characterised genomes or transcriptomes. 8,880 and 8,132 sequences were assigned to the COG classifications for the *Af* and *Oa* transcriptomes, respectively. Among the 25 COG categories, the cluster for “general prediction only” was the largest group in both transcriptomes (4,037 unigenes and 17.4% for the *Af* transcriptome; 3,133 unigenes and 17.9% for the *Oa* transcriptome). This first cluster is followed by the “translation, ribosomal structure and biogenesis” class in *Amphiura* (2,023 unigenes and 8.7% in *Amphiura* against 1286 unigenes and 7.3% in *Ophiopsila*) and the “Replication, recombination and repair” class in *Ophiopsila* (1373 unigenes and 7.8% in *Ophiopsila* against 1731 unigenes and 7.5% in *Amphiura*). Frequency class estimation (percentage in comparison with the total COG annotation) shows a similar pattern in *Af* and *Oa* transcriptomes. Number of COG annotated unigenes are presented in the S2 Fig.

### Functional classification by KEGG

The Kyoto Encyclopedia of Genes and Genomes (KEGG) Pathway is a collection of pathway maps representing the knowledge on molecular interaction and reaction networks [47–48]. The pathway-based analysis is helpful to further understand the biological functions of genes and their interactions. Based on a comparison against the KEGG database using BLASTX with an E-value threshold of 1e^-5^, 21,931 of the 32,815 annotated *A*. *filiformis* unigenes present significant matches. For *O*.*aranea*, 19,452 of the 29,490 annotated unigenes present significant matches. Both brittle star transcriptomes were assigned to 258 KEGG pathways. For both transcriptomes, among the 5 main categories, metabolism was the largest and, more particularly, “metabolic pathways” was the top one hit pathway for both transcriptomes. The 15 top hits KEGG pathways with highest mapping coverage (number of mapped items/number of total items in a pathway) found in both transcriptomes are listed in Table 3. The functional classification of KEGG provided a valuable resource for investigating specific processes, functions and pathways in brittle stars. Multiple metabolism-related pathways are present in the top hits including focal adhesion, regulation of cytoskeleton or purine and pyrimidine metabolism pathways. Several “anthropocentric” pathways (bile secretion, vascular smooth muscle contraction, cardiac muscle contraction) are cited but are supposed to be linked to more general functions such as enzymatic lysis and muscle contraction. Three “Environmental information processing” pathways–relative to “neuroactive ligand-receptor interaction”, “ECM-receptor interaction” and “MAPK signalling pathway” are also listed in the top hits.

**Table 3:** KEGG pathways top hits in the arm transcriptomes of *Amphiura filiformis* and *Ophiopsila aranea*.

	**Pathways**	***Amphiura* unigenes**	**KEGG classification**
1	**Metabolic pathways**	3117 (14.2%)	Metabolism
2	**Focal adhesion**	822 (3.8%)	Cellular Processes
3	**Regulation of actin cytoskeleton**	779 (3.6%)	Cellular Processes
4	**Purine metabolism**	712 (3.2%)	Metabolism
5	**Spliceosome**	697 (3.9%)	Genetic Information Processing
6	**Neuroactive ligand-receptor interaction**	631 (2.9%)	Environmental Information Processing
7	**RNA transport**	628 (2.9%)	Genetic Information Processing
8	**Bile secretion**	539 (2.5%)	Organismal systems
9	**ECM-receptor interaction**	531 (2.4%)	Environmental Information Processing
10	Pyrimidine metabolism	527 (2.4%)	Metabolism
11	Phagosome	523 (2.4%)	Cellular Processes
12	**Endocytosis**	511 (2.3%)	Cellular Processes
13	Vascular smooth muscle contraction	494 (2.3%)	Organismal systems
14	**MAPK signaling pathway**	491 (2.2%)	Environmental Information Processing
15	**Ubiquitin mediated proteolysis**	480 (2.2%)	Genetic Information Processing
	**Pathways**	***Ophiopsila* unigenes**	**KEGG classification**
1	**Metabolic pathways**	2797 (14.38%)	Metabolism
2	**Focal adhesion**	776 (3.99%)	Cellular Processes
3	Pathways in cancer	749 (3.85%)	Human Diseases
4	**Neuroactive ligand-receptor interaction**	684 (3.52%)	Environmental Information Processing
5	Huntington's disease	631 (3.24%)	Human Diseases
6	**Regulation of actin cytoskeleton**	600 (3.09%)	Cellular Processes
7	**Purine metabolism**	582 (2.99%)	Metabolism
8	Epstein-Barr virus infection	568 (2.92%)	Human Diseases
9	**Spliceosome**	555 (2.85%)	Genetic Information Processing
10	**MAPK signaling pathway**	509 (2.62%)	Environmental Information Processing
11	**RNA transport**	495 (2.55%)	Genetic Information Processing
12	**ECM-receptor interaction**	485 (2.49%)	Environmental Information Processing
13	**Ubiquitin mediated proteolysis**	483 (2.48%)	Genetic Information Processing
14	**Bile secretion**	476 (2.45%)	Organismal systems
15	**Endocytosis**	470 (2.42%)	Cellular Processes

Pathways common to both transcriptomes are indicated in bold.

Several pathways associated to light perception were detected including “phototransduction Ko04744” (0.51% in *Amphiura*, 0.54% in *Ophiopsila*) and “phototransduction fly Ko04745” (0.73% in *Amphiura*, 0.63% in *Ophiopsila*). KEGG pathways associated to circadian rhythms were also detected in both species: “circadian rhythm–fly Ko04711” (0.11% in *Amphiura*, 0.12% in *Ophiopsila*) and “circadian rhythm–mammals Ko04710” (0.13% Ophiopsila, 0.13% *Amphiura*). Percent values correspond to the percent of the total amount of unigenes annotated with the KEGG database. Noteworthily, one cryptochrome sequence was detected in both species.

### Candidate opsin transcripts in *O*. *aranea*

We recently identified 13 opsin genes in a draft genome of the brittle star *A*. *filiformis* and the *Af* transcriptome described in the present paper was also previously screened for the presence of opsins. Three opsin mRNAs were discovered in the adult transcriptome of *A*. *filiformis*: one r-opsin (Af-opsin 4.5), one neuropsin (Af-opsin 8.2), and one basal-branch opsin (Af-opsin 2) [29].

Nothing is currently known about a possible opsin-based photoreception in the brittle star *O*. *aranea*. In order to identify opsin mRNAs in *O*. *aranea* arms, homologous sequences to reference opsin gene sequences were searched in the *Oa* transcriptome (tBLASTn, BLASTx). Reciprocal BLAST search analyses allowed to identify transcripts corresponding to 2 different opsins (see S4 Table for blast results): one sequence (Oa-opsin 4; [Unigene64683]) similar to the r-opsin 4 of *S*. *purpuratus* (43% of similarity) and one sequence (Oa-opsin 7; [CL2625.Contig1]) similar to the RGR opsin 7 of *S*. *purpuratus (39% of similarity)*.

**Fig 9:**
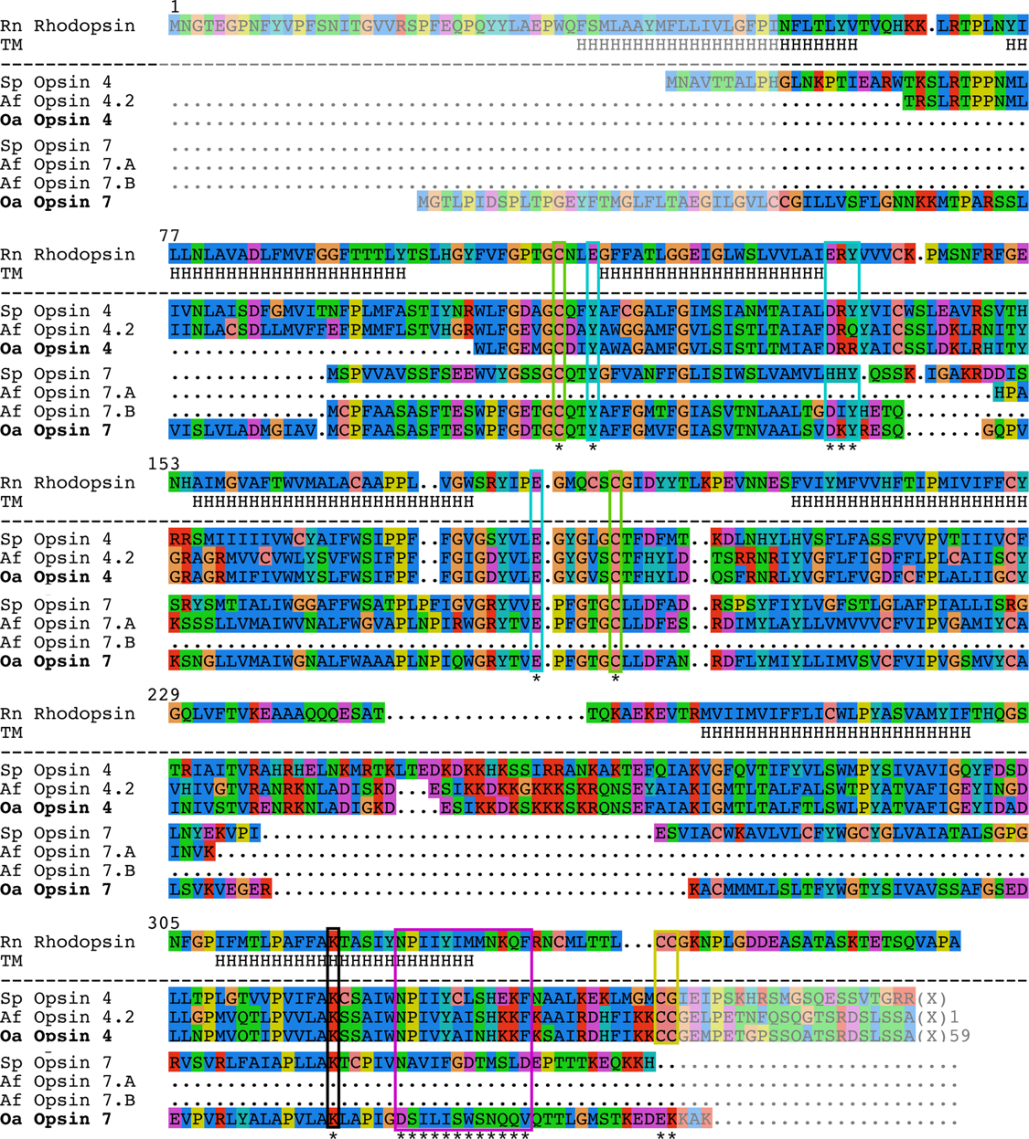
Deduced amino acid sequences of *Ophiopsila aranea* opsins (names in bold in the Fig) aligned with closest *Strongylocentrotus purpuratus* and *Amphiura filiformis* opsins and *Rattus norvegicus* rhodopsin. Non-aligned N-terminus and C-terminus ends are cleared up. Transmembrane alpha-helices of Rn rhodopsin are indicated with H. Opsin-specific residues are marked with *. The lysine residue involved in the Schiff base in the position 296 of the *R*. *norvegicus* rhodopsin is framed in black. Two cysteine residues potentially involved in a disulfide bond are framed in green (positions equivalent to C110 and C187 in *R*. *norvegicus* rhodopsin). A potential palmitoylation motif composed of two contiguous cysteine residues (positions equivalent to C322 and C323 in *R*. *norvegicus* rhodopsin) is framed in yellow at the C-terminus. The tyrosine residue (Y) in position equivalent to the glutamate counterion E113 in *R*. *norvegicus* rhodopsin, glutamate counterion candidate E181 and DRY-type tripeptide motif (E134/R135/Y136 in *R*. *norvegicus* rhodopsin) present at the top of the III TM are framed in blue [85–86]. The pattern “NPxxY(x)6F” (position 302–313 of the *R*. *norvegicus* rhodopsin sequence) is framed in purple. Alignment edited in strap software (http://www.bioinformatics.org/strap/) and in SeaView 4.2.12.

**Fig 10:**
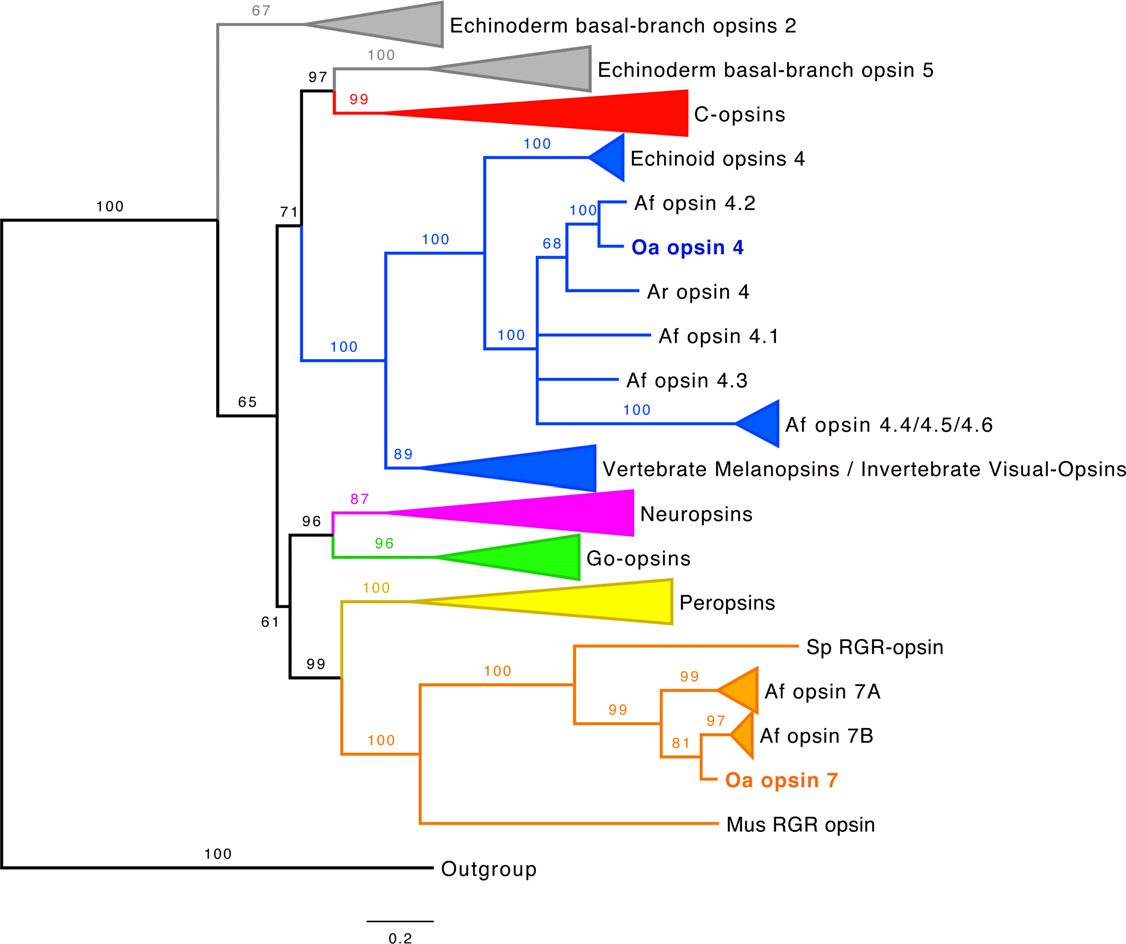
Phylogenetic analysis of representative echinoderm opsins, including new *Ophiopsila aranea* opsins. Sequences cluster into significantly supported subfamilies in Bayesian (illustrated tree) and Maximum Likelihood analyses (see S3 Fig). Branch support values are indicated at important branching points. Branch length scale bar indicates the relative amount of amino acid changes. Sequences are color-coded to indicate their belonging to classical opsin subfamilies (c-opsin in red, r-opsins in blue, neuropsins in pink, Go opsins in green, peropsins in yellow, RGR opsins in orange).

As opsins present high similarities to non-opsin GPCR receptors, a « Blast/reciprocal blast » strategy search should be considered as insufficient to identify *bona fide* opsins. The confirmation of the identification was performed by aligning opsin-like sequences to reference opsins to highlight amino acid residues involved in the opsin function. The complete annotated alignment is presented in the Fig 9, with the description of the *Oa*-opsins (see also S3 File for phy format alignment). Using protein alignment, opsin-specific residues were pinpointed in *Oa*-opsin 4 and *Oa*-opsin 7 (Fig 9). The opsin-specific lysine residue involved in the Schiff Base formation was identified in both predicted proteins. Two cysteine residues potentially involved in a disulfide bond (C110 and C187 in *Rattus norvegicus* rhodopsin) were identified in both *Ophiopsila* opsins. A potential palmitoylation motif composed of two contiguous cysteine residues (C322 and C323 in *R*. *norvegicus* rhodopsin) was highlighted in *Oa*-opsin 4 at the C-terminus. The tyrosine residue (Y) in position equivalent to the glutamate counterion E113 in *R*. *norvegicus* rhodopsin, glutamate counterion candidate E181 and DRY-type tripeptide motif (E134/R135/Y136 in *R*. *norvegicus* rhodopsin) present at the top of the III TM [85–86] were all identified in both *Ophiopsila* predicted opsins. The pattern “NPxxY(x)_6_F” (position 302–313 of the *R*. *norvegicus* rhodopsin sequence) is specific to opsin which have the ability to link with a G-protein [87] and was identified in *Oa*-opsin 4. The “HxK” motif, classically observed in r-opsins [88–89], is present in *Oa*-opsin4. This amino acid triad (in the equivalent position 310–312 in the *R*. *norvegicus* rhodopsin) belongs to the pattern NPxxY(x)_6_F. To confirm the grouping of these sequences to the opsin subfamilies, and to estimate their similarity and homology with known echinoderm opsins, Bayesian phylogenetic and maximum likelihood analyses were performed. The opsin Bayesian phylogenetic tree is presented in Fig 10. Maximum likelihood tree is presented in S3 Fig (Both corresponding tree files are included as supplementary files: S4 and S5 Files). In both cases, the phylogenetic position of *Oa* opsin 4 and 7 are clearly defined in the echinoderm opsin tree within rhabdomeric and RGR opsin groups.

### Candidate transcripts related to phototransduction pathways in brittle star transcriptomes

**Fig 11:**
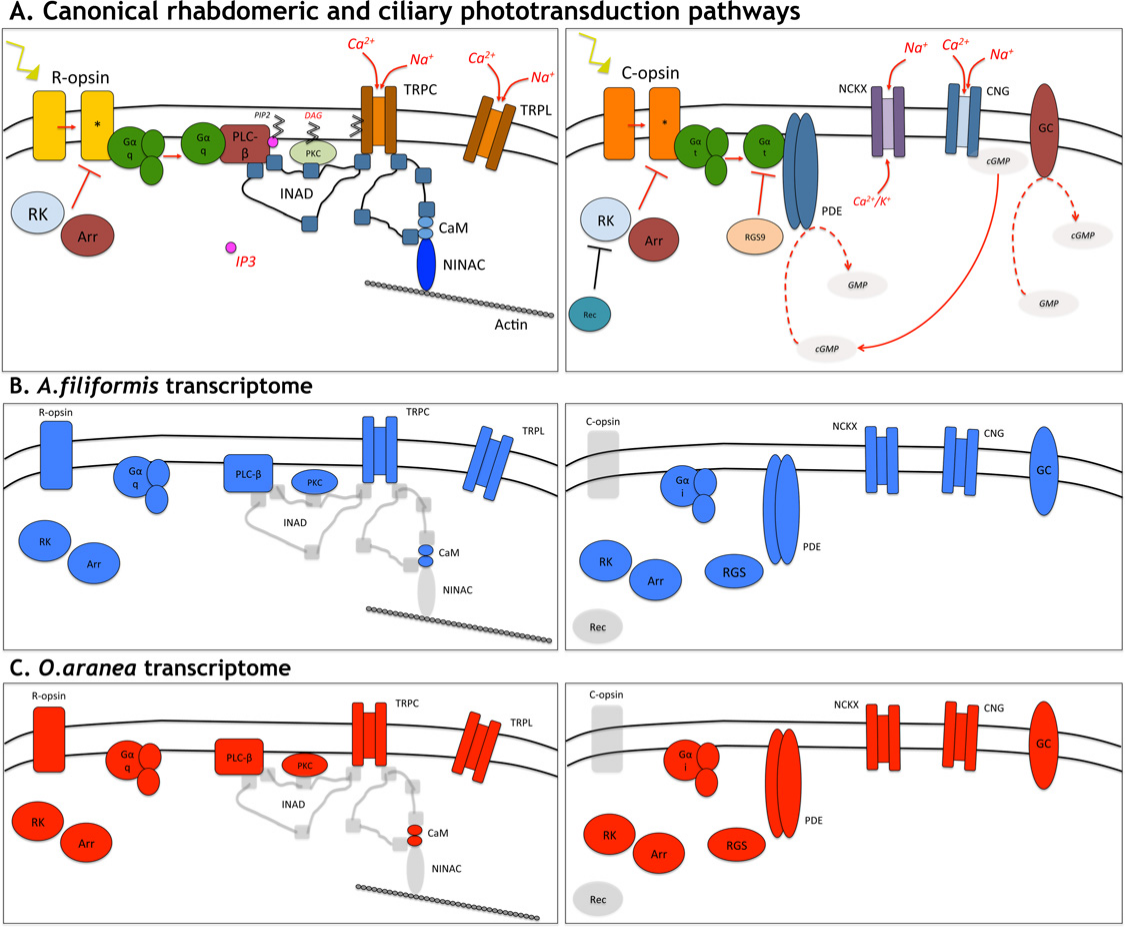
**(A) Schematic drawing of canonical phototransduction signalling pathways downstream of r- and c-opsins. (B) Potential molecular actors highlighted in the arm transcriptome of *Amphiura filiformis*. (C) Potential molecular actors highlighted in the arm transcriptome of *Ophiopsila aranea*.** Actors absent from *Af* and *Oa* transcriptome are cleared up. Actors for which a close homologous is present in *Af*/*Oa* transcriptome are indicated in blue/red.

Phototransduction is a biochemical process by which the photoreceptor cells generate electrical signals in response to captured photons [65]. Two main photransduction cascades characterise rhabdomeric and ciliary photoreceptors in metazoans [90]. Vertebrate ciliary photoreceptors, classically associated with vertebrate eyes, rely on a phototransduction cascade that includes a ciliary opsin, a Gt protein, a phosphodiesterase and cyclic nucleotide gated ion channels (Fig 11A). This cascade starts with the absorption of photons by the photoreceptive c-opsins. Vertebrate c-opsin activation triggers the hydrolysis of cGMP by activating a transducing phosphodiesterase 6 (PDE6) cascade, which results in the closure of the cGMP-gated cation channels (CNG) in the plasma membrane and, therefore, in membrane hyperpolarisation [65]. The hyperpolarisation of the membrane potential of the photoreceptor cell modulates the release of neurotransmitters to downstream cells. The GTP-binding transducin alpha subunit is deactivated through a process that is stimulated by RGS9 (Regulator of G-protein signaling 9). This classical phototransduction is found in rod and cone photoreceptors of vertebrates however some other secondary ciliary phototransduction pathways were described in various organisms including molluscs and jellyfish [90]. Those variations of the canonical pathway primarily result in the use of a different G alpha protein (Gs, Go, Gi). However the large majority of the ciliary phototransduction pathways affect the cGMP signaling pathways [90].

Rhabdomeric photoreceptors, classically associated with invertebrate eyes, employ a cascade involving r-opsins, Gq-protein, phospholipase C and transient receptor potential ion channels (TRP, TRPL) (Fig 11B). Visual signaling is initiated with the activation of the r-opsin by light. Upon absorption of a photon, the opsin chromophore is isomerised, which induces a structural change that activates the opsin. This photoconversion activates a heterotrimeric Gq protein via GTP-GDP exchange, releasing the Gα-q subunit. The Gα-q activates the phospholipase C (PLC), generating IP_3_ (inositol 1,4,5-triphosphate) and DAG (diacylglycerol) from PIP_2_ (phosphatidylinositol). This reaction leads to the opening of cation-selective channels (TRP and TRPL) and causes the depolarisation of the photoreceptor cell membrane. In the canonical *Drosophila* pathway, several transduction proteins are coordinated by a polymer of INAD scaffold proteins [65,90–91]. Again, some rare variations were described with the use of a different G-protein (Gi, Go) [92–93].

In both phototransduction pathways, recovery involves the deactivation of the light-activated intermediates. Photolysed c- and r-opsin are phosphorylated by rhodopsin kinases and subsequently capped-off by arrestins (Fig 11) [64,66,90].

Analysis of the unigenes retrieved from the two brittle star transcriptomes revealed transcripts encoding proteins with high similarities to the key components of visual transduction cascades. Genes encoding proteins involved in both the activation and deactivation of the cascades were identified. A reciproqual BLASTx analysis demonstrated that several predicted proteins were highly similar to the phototransduction actors. The identifier and the E-value of the top hit are listed in the S5 Table. Blasts hits with significant E-values strongly indicate homologous proteins. Putative candidates for which the reciprocal BLAST analysis indicated a strong homology with classical phototransduction actors are highlighted in Fig 11B and 11C. Conversely, some candidate proteins retrieved by the first blast search were not unambiguously identified by the reciprocal blast. This is the case for the *Drosophila*-specific InaD scaffold protein which gave a hit to « Lin-7 protein homologue » in both transcriptomes. Overall, the results indicate that both ciliary and rhabdomeric phototransduction actors are expressed in the arms of *A*. *filiformis* and *O*. *aranea* indicating these two pathways should be active. Considering the ciliary phototransduction, however, as no ciliary opsin were identified, we cannot confirm the “active status” of this pathway.

## Conclusion

Until recently, the vast majority of molecular studies on echinoderms have focused on sea urchins, partly because of the lack of extensive genomic resources in the other echinoderm classes. However, in non-classical model species, NGS recently allowed to generate molecular data and therefore to detect new genes and analyse their expression. In this study, we report the sequencing and analysis of arm transcriptomes from two European brittle star species, *A*. *filiformis and O*. *aranea*.

The substantial amount of transcripts obtained will certainly accelerate the understanding of many biological mechanisms in brittle stars and echinoderm in general. Whole tissue libraries imply the high expression of some essential “house-keeping” genes. The most expressed transcripts highlighted in both transcriptomes indeed correspond to some of these housekeeping genes. Nevertheless, the Illumina sequencing technology, which allows high depth sequencing and coverage, help to identify genes expressed in a potentially restricted spatial pattern like opsins. The expression analyses performed on opsins and on photoreceptor markers made it possible to highlight several potential actors of rhabdomeric and ciliary phototransduction pathways in brittle star arm tissues, confirming the ability of the brittle star to perceive light without eye *stricto sensu*. It has to be reminded that, with the exception of opsins, phototransduction actors are not exclusively restricted to opsin-based transduction and are involved in other GPCR transduction pathways.

Opsin genes were recently identified in *A*. *filiformis* [29]. Partial opsin transcripts corresponding to three of these genes were detected in the transcriptome data generated in the present study: one rhabdomeric opsin (Af-opsin 4.2), one basal-branch opsin 2 (Af-opsin2) and one neuropsin (Af-opsin 8). Here we completed this study by the detection of actors of both rhabdomeric and ciliary phototransduction pathways. In *O*. *aranea*, two new opsins were detected: one rhabdomeric opsin and one RGR opsin. With the absence of the conserved region involved in the G-protein contact, the RGR-opsin could act as a photoisomerase involved in retinal regeneration as described in vertebrates [94, 95]. The r-opsin is therefore the best and only visual opsin candidate in *O*. *aranea*, as proposed for the r-opsin from sea urchin tube feet [26]. In view of its behaviour, *O*. *aranea* appears to be characterized by a monochromatic colour perception for which the maximal sensitivity is in the green wavelength. A negative phototactic response was indeed observed for green illumination (λmax **=** 515nm) while a non-directional phototaxis was highlighted for blue illumination (λmax **=** 464nm). The detected *Oa* r-opsin could therefore be maximally sensitive to green light, and the blue colour perception could be assimilated to a more residual perception due to the relative wide absorption spectrum of opsins (around 100 nm at half-peak width; see e.g. [96–97]).

Considering the expression of highly similar r-opsin genes in both species (Af-opsin 4.2 and Oa-opsin 4), light-mediated behaviour differences between the two brittle stars do not appeared to be linked to differences in opsin diversity or expression. It is however important to pinpoint that faintly expressed opsin or molecular markers could potentially be absent from our dataset considering the read coverage we were using in this tissue-specific (arm) RNA-seq study (50M reads). Further analyses using more reads could help to reach a more complete view of the RNA expressed in the brittle star tissues and help for the discovery of new genes especially when their expression is low.

On the other hand, morphological adaptations such as the stereom microlenses (highlighted in *O*. *aranea* and absent from *A*. *filiformis* dorsal arm plates) could potentially explain the striking differences observed in phototactic responses. Microlens-like structures present on aboral arm plates were indeed shown (i) to be involved in light focalization [34] and (ii) associated to potential underlying photoreceptors [31–32]. *O*. *aranea* is clearly flying away under green illumination indicating a directional phototaxis potentially enhanced by the presence of precited microlens-like structures.

## Supporting Information

S1 Fig Gene coverage estimation using nr database as a reference. (PDF)

S2 Fig COG function classification for unigene sequences from the arm transcriptomes of *Amphiura filiformis* and *Ophiopsila aranea*. (TIFF)

S3 Fig Maximum likelihood phylogenetic analysis of representative echinoderm opsins, including new Ophiopsila aranea opsins. (PDF)

S1 File *Amphiura filiformis* unigenes (fasta). (ZIP)

S2 File *Ophiopsila aranea* unigenes (fasta). (ZIP)

S3 File Trimmed opsin alignment used for phylogenetic analysis (phy file) (PHY)

S4 File Tree file of MrBayes phylogenetic analysis. (TRE)

S5 File Tree file of Maximum likelihood phylogenetic analysis. (TREE)

S1 Table List of the reference opsins used for blast (A) and phylogenetic analyses (A,B). Echinoderm opsins are presented in bold.
(XLSX)

S2 Table Reference key genes of rhabdomeric and ciliary phototransduction pathways. InaD: inactivation no afterpotential, TRP: transient receptor potential, TRPL: transient receptor potential-like, PDE: phophodiesterase, RGS9: regulator of G-protein signaling 9, NCKX: Na/K/Ca exchanger 1 isoform 2, cGMP: cyclic guanosine monophospate, GC: guanylate cycle, CNG: cGMP gated channel, SU: subunit, Arr: arrestin, RK: rhodopsin kinase. *Recently, Gi-proteins were also shown to interact with r-opsins/melanopsins [93].
(XLSX)

S3 Table Statistical analyses relative to the behavioural study of the light perception in *Ophiopsila aranea*. (XLSX)

S4 Table BLASTP results and characteristics of the *Oa*-opsin genes. DNA: DNA fragment size, Pep: peptide size prediction, TM: number of predicted transmembrane helix, MW: predicted molecular weight, Acc.: accession, Q.C.: query coverage, E val: E value, Id: identity. Reference numbers are the following: *Oa*-opsin 4 [Unigene64683], *Oa*-opsin 7 [CL2625.Contig1].
(XLSX)

S5 Table Search for key phototransduction genes in the adult transcriptome of *Amphiura filiformis* and *Ophiopsila aranea*. Homologues to Gq and Gt/o/i phototransduction cascade components and their reciprocal best BLAST hit. For each query protein, the corresponding *A*. *filiformis* and *O*. *aranea* unigene are listed with the E-value of the top blast result. The *A*. *filiformis* and *O*. *aranea* proteins were then used as query in a reciprocal best BLAST search of the non-redundant protein database (NBCI) and the top result is listed along with the E-value of the blast result. Reference sequence accessions are listed in the S2 Table. Reciprocal best BLAST hits to proteins with annotations that closely correspond to the query proteins are in bold and indicate good candidates of phototransduction actors. Arr: arrestin, cGMP: cyclic guanosine monophospate, GC: guanylate cycle, CNG: cGMP gated channel, ID: identification number, InaD: inactivation no afterpotential, NCKX: Na/K/Ca exchanger 1 isoform 2, PDE: phophodiesterase, RGS9: regulator of G-protein signaling 9, RK: rhodopsin kinase, SU: subunit, TRP: transient receptor potential, TRPL: transient receptor potential-like. Species ID; *A*. *carolinensis*: *Anolis carolinensis*, *B*. *taurus*: *Bos taurus*, *H*. *sapiens*: *Homo sapiens*, *S*. *kowaleskii*: *Sacoglossus kowalevski*.
(XLSX)
