# Supplementary figures and images for: De Novo Adult Transcriptomes of Two European Brittle Stars: Spotlight on Opsin-Based Photoreception
Source: PLoS One. 2016 Apr 27;11(4):e0152988. doi: 10.1371/journal.pone.0152988 (PMC4847921; doi:10.1371/journal.pone.0152988)

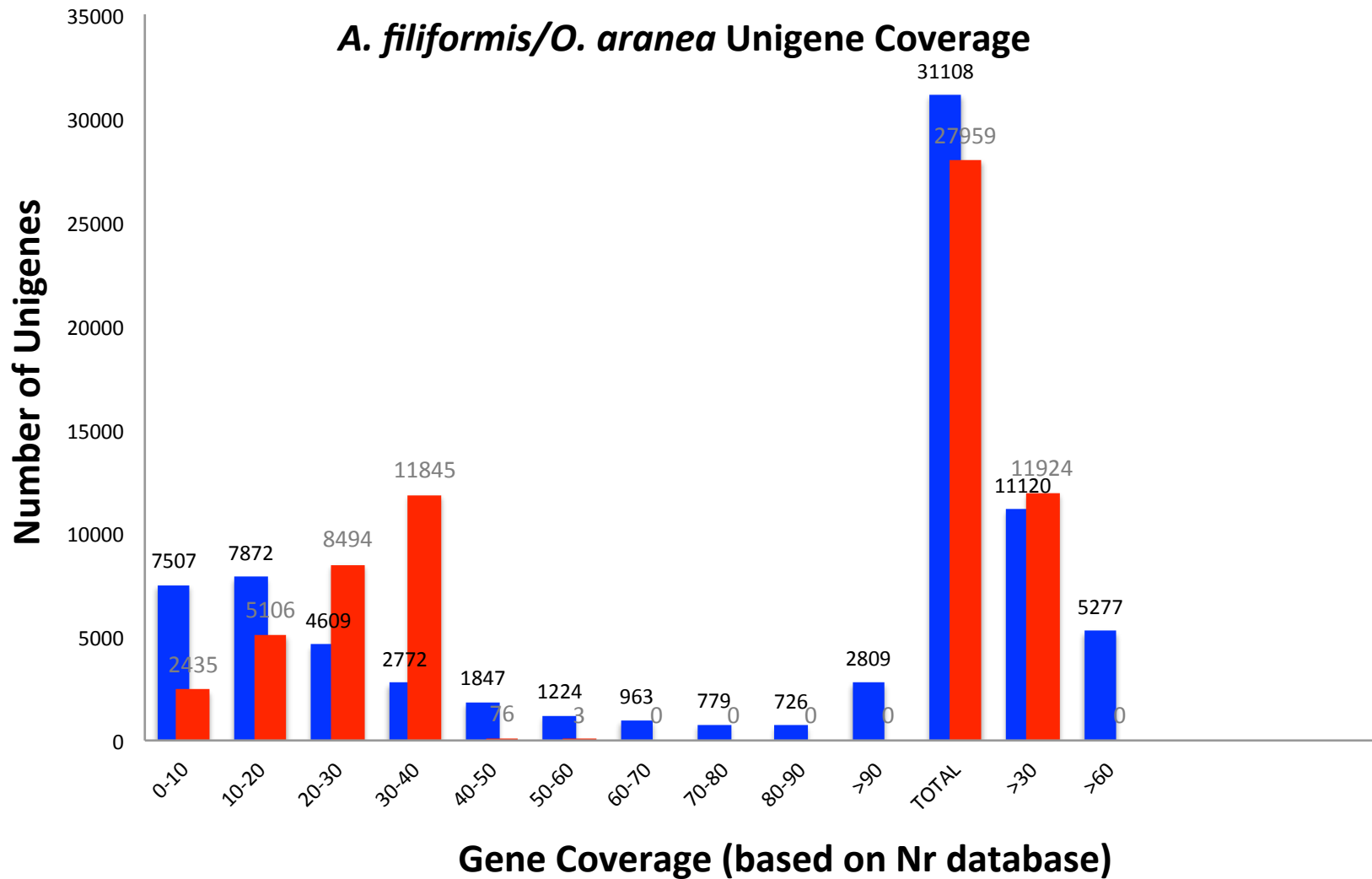

Supplement: S1 Fig — (PDF) [file pone.0152988.s001.pdf]

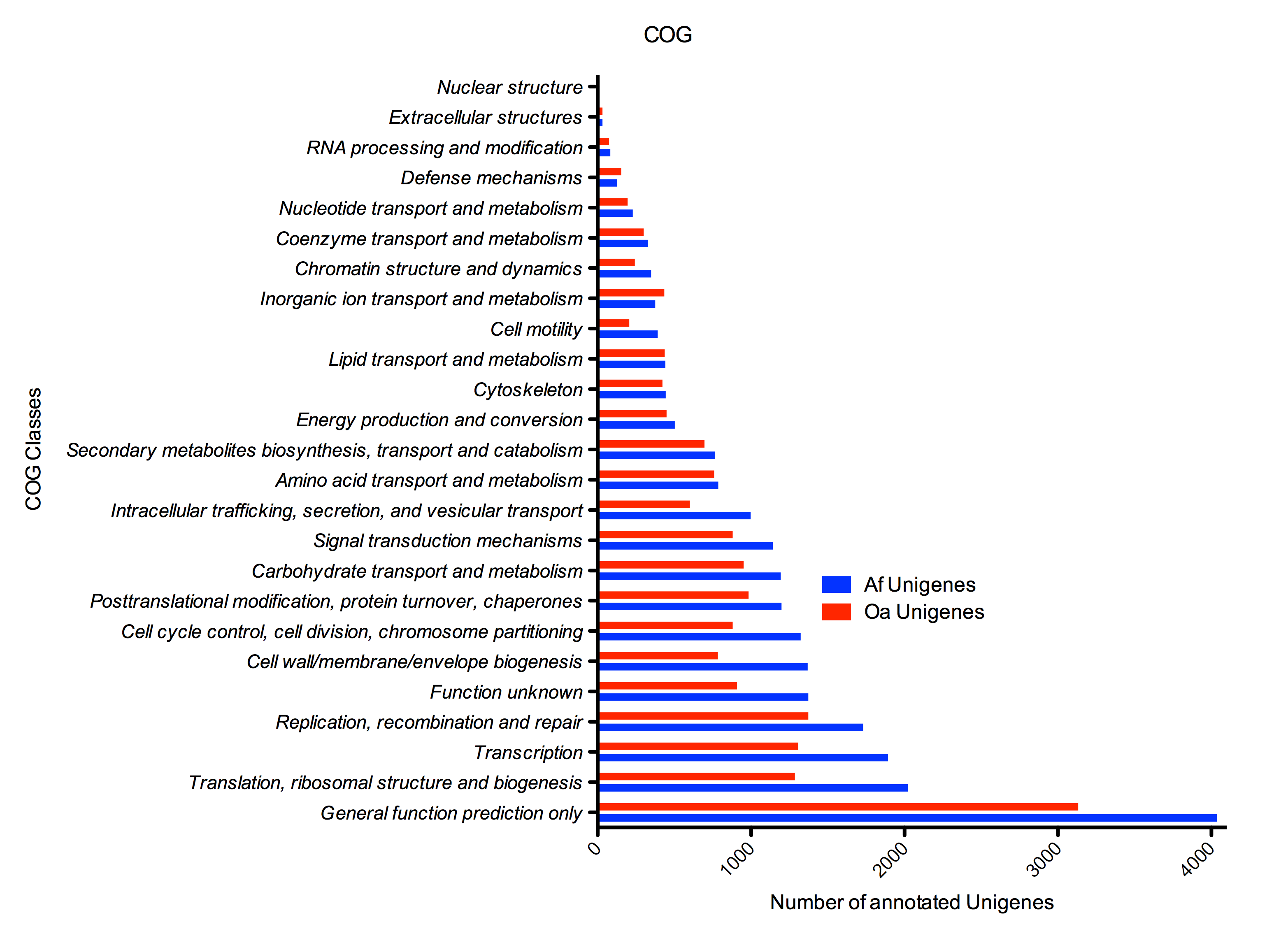

Supplement: S2 Fig — (TIFF) [file pone.0152988.s002.tiff]

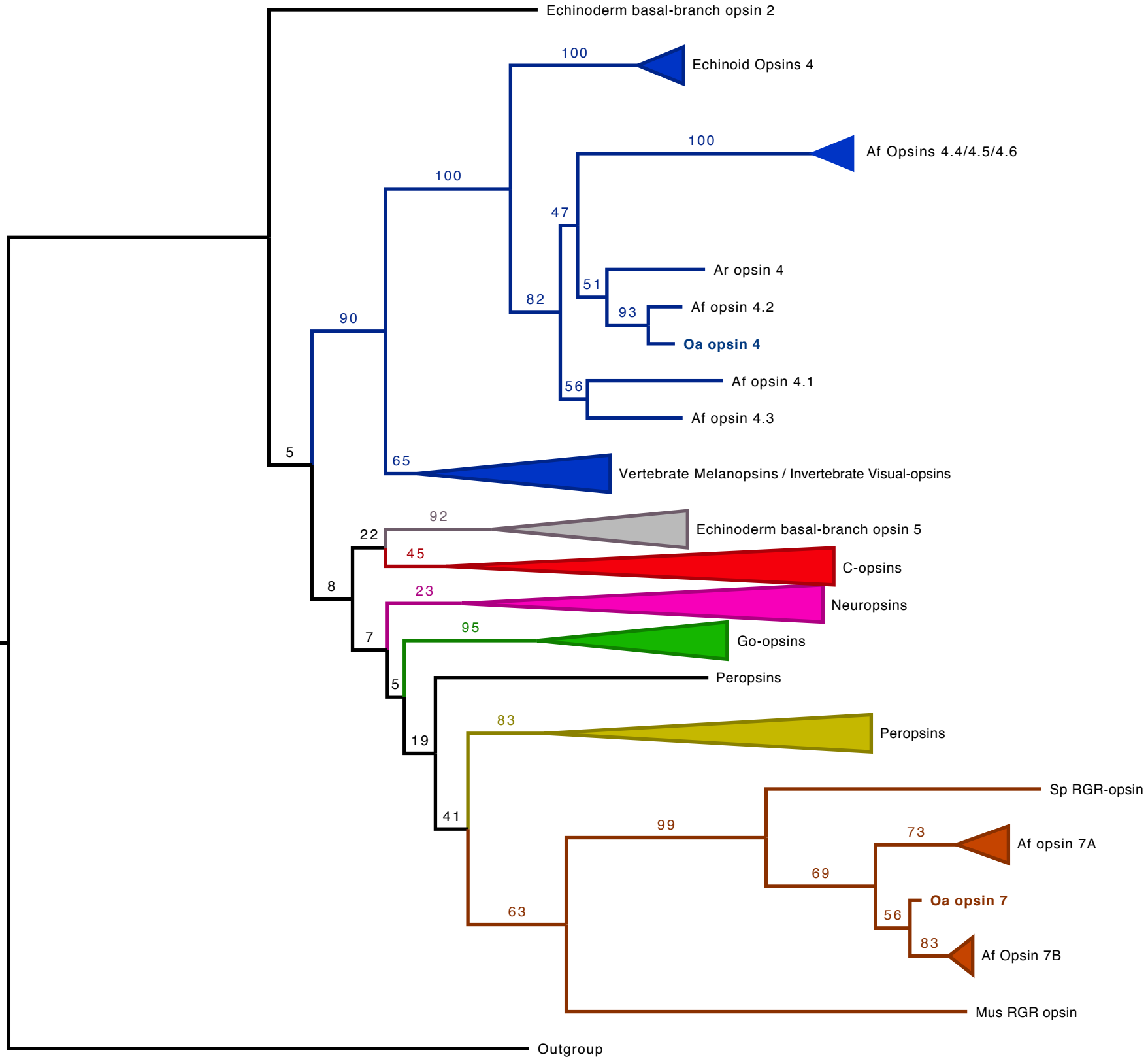

Supplement: S3 Fig — (PDF) [file pone.0152988.s003.pdf]
